# Supplementary figures and images for: Dissecting antibody-dependent enhancement modulation by Fc-modified cross-neutralizing human monoclonal antibody
Source: PeerJ. 2025 Nov 19;13:e20329. doi: 10.7717/peerj.20329 (PMC12640126; doi:10.7717/peerj.20329)

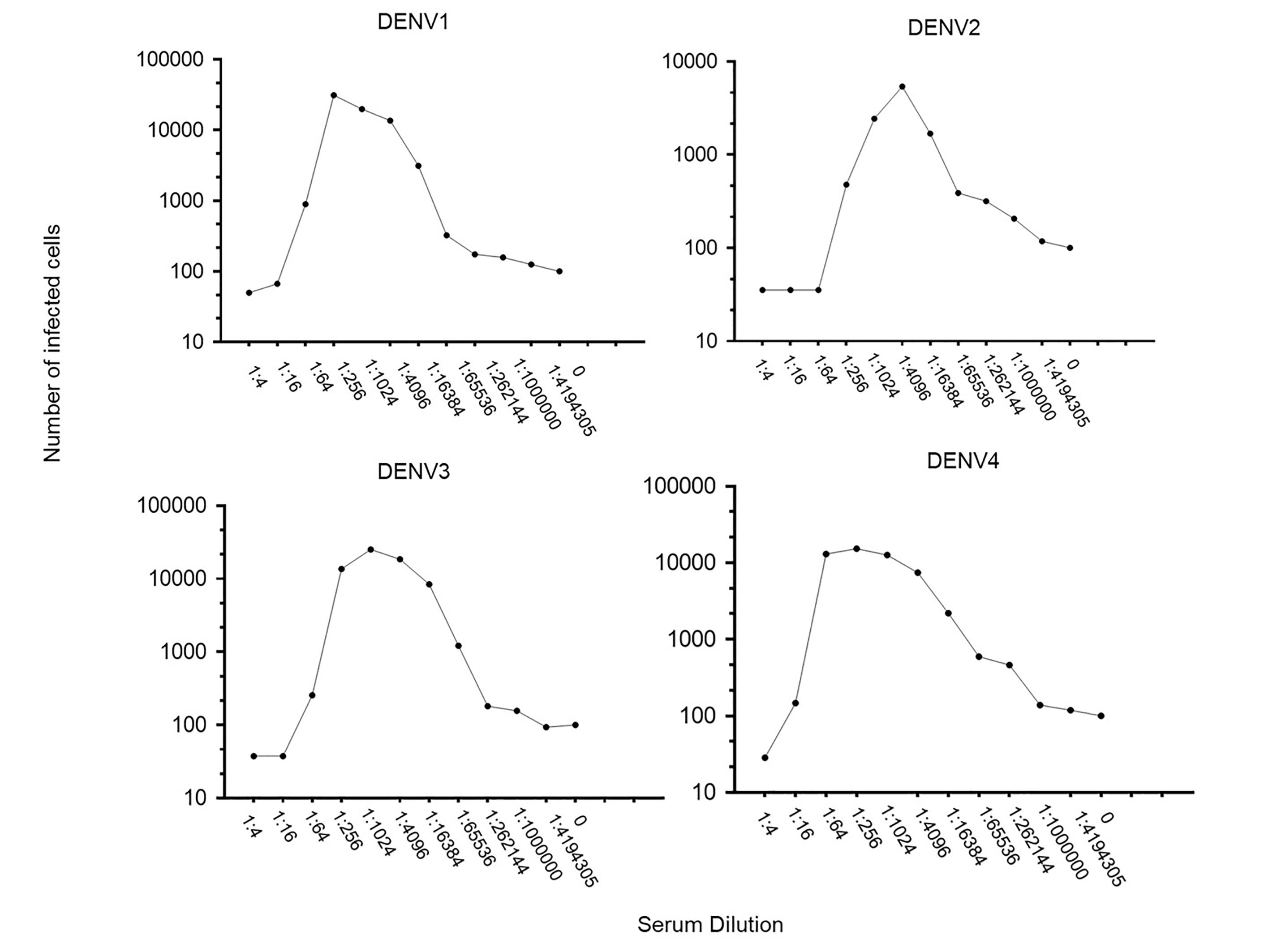

Supplement: Supplemental Information 7 — Serial dilutions of serum from a patient with acute DENV-2 infection were incubated with K562 cells to assess infection rates. The optimal dilutions yielding the greatest enhancement of dengue virus serotypes were 1:4000 for DENV-1 and DENV-2, and 1:1000 for DENV-3 and DENV-4. [file peerj-13-20329-s007.png]
